# Integrated Communication System: Gesture and Language Acquisition in Typically Developing Children and Children With LD and DLD

**DOI:** 10.3389/fpsyg.2020.00118

**Published:** 2020-02-04

**Authors:** Carina Lüke, Ute Ritterfeld, Angela Grimminger, Katharina J. Rohlfing, Ulf Liszkowski

**Affiliations:** ^1^Faculty of Arts and Humanities, Psycholinguistics, Paderborn University, Paderborn, Germany; ^2^Department of Language and Communication, School of Rehabilitation Sciences, TU Dortmund University, Dortmund, Germany; ^3^Developmental Psychology, Institute of Psychology, University of Hamburg, Hamburg, Germany

**Keywords:** language acquisition, developmental language disorder, language delay, pointing, iconic gestures

## Abstract

Gesture and language development are strongly connected to each other. Two types of gestures in particular are analyzed regarding their role for language acquisition: pointing and iconic gestures. With the present longitudinal study, the predictive values of index-finger pointing at 12 months and the comprehension of iconic gestures at 3;0 years for later language skills in typically developing (TD) children and in children with a language delay (LD) or developmental language disorder (DLD) are examined. Forty-two monolingual German children and their primary caregivers participated in the study and were followed longitudinally from 1;0 to 6;0 years. Within a total of 14 observation sessions, the gestural and language abilities of the children were measured using standardized as well as *ad hoc* tests, parent questionnaires and semi-natural interactions between the child and their caregivers. At the age of 2;0 years, 10 of the 42 children were identified as having a LD. The ability to point with the extended index finger at 1;0 year is predictive for language skills at 5;0 and 6;0 years. This predictive effect is mediated by the language skills of the children at 3;0 years. The comprehension of iconic gestures at 3;0 years correlates with index-finger pointing at 1;0 year and also with earlier and later language skills. It mediates the predictive value of index-finger pointing at 1;0 year for grammar skills at 5;0 and 6;0 years. Children with LD develop the ability to understand the iconicity in gestures later than TD children and score lower in language tests until the age of 6;0 years. The language differences between these two groups of children persist partially until the age of 5;0 years even when the two children with manifested DLD within the group of children with LD are excluded from analyses. Beyond that age, no differences in the language skills between children with and without a history of LD are found when children with a manifest DLD are excluded. The findings support the assumption of an integrated speech–gesture communication system, which functions similarly in TD children and children with LD or DLD, but with a time delay.

## Introduction

Gesture and language acquisitions are related to each other: From the end of their first year of life infants are using gestures and oral language to understand others and to communicate with them, with both serving as means of communication. In this line, McNeill (1992) and Kendon (2004) propose that gestures and oral language form an integrated communication system. Findings from studies with children and adults producing utterances with semantic supplementary and reinforcing gesture-word-combinations (e.g., Eriksson, 2018) as well as results indicating lower performances in gesture tasks in language-impaired populations compared to typically developed speakers (e.g., Botting et al., 2010; Wray et al., 2016) support the assumption of such an integrated communication system. However, based on findings that gestures facilitate word retrieval in language impaired adults, other authors (e.g., Hadar et al., 1998) argue for two separate systems.

The empirical research, especially in the past two decades, provides a multitude of findings about the relationship between gestures and oral language in typically developing (TD) children and children with a language delay or even disorder (e.g., Botting et al., 2010; Colonnesi et al., 2010; Mainela-Arnold et al., 2014; Wray et al., 2017; Iverson et al., 2018). Two types of gestures and their role in language acquisition are the focus of research. These two types are *pointing gestures* and *iconic gestures*.

Pointing gestures are one of the first means of intentional communication and a subtype of deictic gestures which are used to refer to something (mostly) in immediate surroundings (Bates, 1976; Liszkowski, 2008). Infants start to use pointing gestures within the second half of their first year of life and often before beginning with word production. First, infants use so called *whole-hand* points, in which the arm and the hand are extended toward a referent, followed by *index-finger points*, in which the arm and the index finger are clearly extended toward a referent (Lock et al., 1990; Liszkowski and Tomasello, 2011; Lüke et al., 2017b). Based on the large number of studies it is undisputable that the production of pointing gestures is a strong predictor for later language skills (for a meta-analysis see Colonnesi et al., 2010 (for recent research see Murillo and Belinchón, 2012; Beuker et al., 2013; Kuhn et al., 2014; Lüke et al., 2017a, 2019; Salo et al., 2018). Yet, there is some debate about the specific aspect of pointing gestures responsible for this predictive value. The following features are analyzed and discussed: 1. Onset or ability to produce pointing gestures at a certain age (Lüke et al., 2017a, 2019; McGillion et al., 2017); 2. Number of pointing gestures (Beuker et al., 2013; Kuhn et al., 2014; Lüke et al., 2017b); 3. Intention of pointing gestures (Colonnesi et al., 2010; Lüke et al., 2017a; Salo et al., 2018); 4. Hand shape of pointing gestures (Liszkowski and Tomasello, 2011; Murillo and Belinchón, 2012; Lüke et al., 2017a, b, 2019); 5. Number of referents (Rowe et al., 2008; Rowe and Goldin-Meadow, 2009); and 6. Combination with words (Desmarais et al., 2008; Fasolo and D’Odorico, 2012; Murillo and Belinchón, 2012; Cartmill et al., 2014; Igualada et al., 2015; Grimminger et al., 2016; Eriksson, 2018). While previously the intention of pointing gestures seemed to be essential for their predictive value (Colonnesi et al., 2010), more recent research indicates that the onset of pointing gestures, the hand shape, the combination of pointing gestures and words, and the number of different referents are better predictors for later language skills (Rowe and Goldin-Meadow, 2009; Lüke et al., 2017a, 2019; Eriksson, 2018). Lüke et al. (2017a) showed that at the age of 12 months the hand shape and not the intention of pointing gestures is responsible for the predictive value. At this age index-finger points, but not hand points, are strongly predictive for later language skills regardless of their intention (imperative vs. declarative). The strong predictive value of pointing gestures has mostly been analyzed within the first 3 years of life. A few studies show that early pointing gestures are predictive for language skills until the age of 4;0–4;6 (years; months) (Cartmill et al., 2014; Lüke et al., 2019). Beyond that age, no studies can be found (cf. Rohlfing, 2019).

The strong relation between early pointing gestures and language skills is not only found in TD children, but also in children with high risk for developmental disorders, such as: siblings of children with autism spectrum disorder (Baron-Cohen et al., 1992; Parladé and Iverson, 2015; Iverson et al., 2018; Sansavini et al., 2019), extremely preterm infants (Benassi et al., 2018; Sansavini et al., 2019), and children with Down Syndrome (te Kaat-van den Os et al., 2015). However, there are hardly any studies about early pointing behavior within a comparatively large group of children, namely children with language delay (LD) and developmental language disorder (DLD). Children with LD, often called late talkers, are children between the age of 2;0 and 2;11 with limited language skills. Often, their productive vocabulary is small (<50 words) and they do not produce two-word-combinations (Desmarais et al., 2008). Around 15–20% of all children display a LD (Reilly et al., 2007). Receptive language deficits, a family history of language disorders, and a low maternal level of education are negative predictors for later language skills within this group (Desmarais et al., 2008; Reilly et al., 2018). The prognosis for an individual child with LD is rather poor. Depending on the outcome variable and the inclusion criteria of the children within studies, around 20–80% of the children with LD catch up in their language skills until their third birthday (Rescorla et al., 1997; Dale et al., 2003; Miniscalco et al., 2005). With the remaining children LD persists and eventually manifests as DLD. Children with DLD have significant language problems with a substantial “impact on everyday social interactions or educational progress” (Bishop et al., 2017, p. 1070), which are likely to persist into middle childhood and beyond, but without any known differentiating condition such as for example, Down Syndrome (Bishop et al., 2017). About 7–8% of preschool-children are affected by such significant language problems (Tomblin et al., 1997; Norbury et al., 2016), which are high risk factors for educational success, mental health, and a fulfilling social life into adulthood (Tomblin et al., 2003; Clegg et al., 2005; Law et al., 2009).

Longitudinal studies with siblings of children with autism spectrum disorder often comprise three different outcome groups: Siblings without developmental disorders, siblings with LD, and siblings with autism spectrum disorder (Parladé and Iverson, 2015; Iverson et al., 2018). From these studies we know that not only the siblings who turned out to be within the autism spectrum themselves, but also the siblings with a later diagnosis of LD produce a lower number of gestures early in their development and have a lower rate of initial growth in early gestures than TD children (Parladé and Iverson, 2015; Iverson et al., 2018; Sansavini et al., 2019). For children with LD, Bello et al. (2018) found that conspicuously more of them do not produce declarative pointing gestures compared to TD children. Beyond that, O’Neill and Chiat (2015) and O’Neill et al. (2019) showed that children with a receptive-expressive LD score more poorly in gesture production tasks than children with an expressive-only LD and that the performance in the gesture tasks in turn predicts later language skills. In earlier publications concerning the sample presented here, we found that children who do not point with the extended index finger at 12 months are at higher risk for LD at 2;0 compared to children who do produce index-finger points at 12 months of age (Lüke et al., 2017a). Further, children with typical language development reduce the use of whole-hand points within the second year of life, but children with LD do not, so that at the end of the second year of life, children with LD use more pointing gestures in total than TD children (Lüke et al., 2017b).

Many more studies about iconic gestures in children with LD and DLD have been published. Iconic gestures represent semantic information in their form or movement (e.g., opening and closing the extended index and middle finger to represent scissors). This research focuses on four different aspects of iconic gestures: (1) Comprehension of the iconicity of iconic gestures (Botting et al., 2010; Wray et al., 2016; Perrault et al., 2019); (2) Production of iconic gestures during specific tasks such as narrative tasks, picture description, or in shared book-reading situations (Iverson and Braddock, 2011; Mainela-Arnold et al., 2014; Lavelli et al., 2015; Lavelli and Majorano, 2016; Wray et al., 2016, 2017); (3) The beneficial effect of iconic gestures for word learning (Ellis Weismer and Hesketh, 1993; Lüke and Ritterfeld, 2014; Vogt and Kauschke, 2017); (4) Adaptation in parental input (Grimminger et al., 2010; Wray and Norbury, 2018). Tolar et al. (2008) showed in their cross-sectional study with TD children that the ability to understand the meaning of iconic gestures even in this group is only slightly developed, with just 14% of the 2;6-year-old children performing above chance. From the age of 3;0 more than half of the children perform above chance, but on average only 46% of all answers are correct at this age. The ability to comprehend the iconicity of the presented gestures increases further, so that the children between 4;6 and 5;0 match 76% of the gestures to the correct picture. The ability to recognize the meaning of iconic gestures at 2;6 and 3;0 is connected to productive lexical skills of the children. If they are able to name a picture, they are more likely to correctly match the iconic gesture to it.

Besides utilizing situational resources for communication, language learning comprises the ability to focus on some relevant aspects of entities. Iconic gestures can capture and refer to these aspects. In a study with 3-year-olds, Mumford and Kita (2014) argue that by viewing particular aspects of verbs (e.g., manner of actions vs. change-of-state) highlighted in gesture, children learned the presented verbs in accordance with those aspects that were encoded in gesture. From the current state of research on learning from iconic gestures, it is reasonable to assume that the ability to comprehend gestures relates to schematization processes that are crucial in conceptualizing events and learning their communicative meanings (Mumford and Kita, 2014; Rohlfing, 2019). Children with DLD seem to need more time for schematizing those processes: Botting et al. (2010) showed that children with DLD scored more poorly on a comprehension task which required them to integrate verbal and gestural information than TD children. The test consisted of a spoken sentence where the last word was expressed via gesture but not verbally. Beyond the poorer scores of the children with DLD, differences in the error patterns between the two groups were also found. Whereas TD children made more verbal language based errors (i.e., they chose a distractor that was semantically correct but only for the verbal part of the message), children with DLD made more gesture based errors by picking a distractor which was semantically correct but only for the gestural part of the message (Botting et al., 2010). These findings were replicated by Wray et al. (2016) and support the hypothesis of an integrated communication system by showing that children with DLD scored more poorly in the gesture comprehension task and relied more on the information which was delivered through gestures.

Findings from studies comparing iconic gesture production in children with DLD and TD children are more conflicting: While some authors found that children with DLD produce more gestures in gesture production tasks than TD children (Iverson and Braddock, 2011; Mainela-Arnold et al., 2014; Lavelli et al., 2015), others found no differences in the number of gestures produced (Botting et al., 2010; Wray et al., 2017), but in the accuracy of their production (Wray et al., 2017). These results also seem to depend on the language skills and age of the comparison group of TD children: In shared book-reading situations, children with DLD produce more gestures in total when compared to age-matched TD children, but a similar number of gestures when compared to language-matched TD children (Lavelli et al., 2015). Although, overall, all groups of children in this study produce mostly pointing gestures, children with DLD produce a higher rate of iconic gestures compared to TD children who are age matched, but not a higher rate in comparison to the younger, language-matched ones (Lavelli et al., 2015).

In another line of studies, the beneficial effect on word learning of iconic gestures in the input is documented in young TD children (e.g., Capone and McGregor, 2005; McGregor et al., 2009) and in children with DLD (Ellis Weismer and Hesketh, 1993; Lüke and Ritterfeld, 2014; Vogt and Kauschke, 2017). These findings are in line with the observation that mothers of children with LD or DLD intuitively adapt their input to their children by providing more pointing and iconic gestures in interactions than mothers of the same-aged TD children (Grimminger et al., 2010; Lavelli et al., 2015; Wray and Norbury, 2018). Lavelli et al. (2015) show again that this higher rate of gestures in maternal input of children with DLD is comparable to the number of gestures produced in maternal input of younger, language-matched TD children.

Taken together, gestures—especially pointing and iconic gestures—are strongly connected to language acquisition. While pointing gestures are mostly analyzed as a predictor variable, the focus on iconic gestures is situated in their comprehension, spontaneous production, and supportive effect on word learning. Little is known about the relation between these two types of gestures and the predictive value of iconic gesture comprehension for further language acquisition. The role of both gesture types is even more significant for children with LD and DLD. With the current study, we therefore investigate (1) whether the predictive value of index-finger pointing at 1;0 persists to language skills beyond the age of 4;0 (cf. Lüke et al., 2019) and, if so, whether children’s language skills during the intervening time period mediate this relation. We expect that the predictive value of index-finger pointing at 1;0 persists until the age of 6;0 years and that this relation is mediated by the language skills between 3;0 and 4;0 years of age. (2) Further, we want to investigate the relation of early index-finger pointing and later iconic gesture comprehension, two gestural parts of an integrated communication system. Based on this assumption of an integrated communication system, we also expect the ability to understand the iconicity of iconic gestures to be predictive for later language skills, too. (3) Since the predictive value of gestural skills is especially important for children with LD and DLD we investigate the developmental pathways of gesture and language skills in children identified as LD at 2;0 until the age of 6;0 and compare their developmental pathways with those of TD children. In addition to differences in language skills, we expect to find also differences in gestural skills between children with and without a LD.

## Materials and Methods

### Participants

Forty-five children and their primary caregivers (96% mothers) participated in this longitudinal study. The data of three children was excluded because they did not participate after the age of 2;6 (2) or because of a chronic otitis media with several effusions (1). The final sample consists of 42 children (24 boys, 18 girls) with a mean age of 12 months and 7 days (*SD* = 15 days) at the beginning of the study. The families were recruited via the pediatricians of the children during their regular medical check-up between the children’s ages of 10 to 12 months. The pediatricians informed the families about the study and asked them if they were interested to participate. The medical personal was encouraged to especially invite families with a sibling or a parent with a history of language disorder to the study in order to increase the number of children with a higher risk of LD (Rice et al., 2009). This effort resulted in 8 of the 42 children being from families with at least one sibling or parent having a history of LD.

All children were raised as monolingual German speakers. Their general development was rated by their pediatricians and measured at the beginning of the study with a standardized test that included cognitive, motor, language, social, and emotional development (*Entwicklungstest für Kinder von 6 Monaten bis 6 Jahren*, ET 6-6 [Developmental test for children aged 6 months to 6 years], Petermann et al., 2008). According to pediatricians as well as standardized test results, all children were developing typically. At 3;6, the non-verbal IQ was measured using the non-verbal IQ-test SON-R (Tellegen et al., 2007). Information about the socioeconomic status (SES) of the family was collected via parent reports. On average, the children were being raised by parents with a rather high educational level and an average household income (compared to the median family income in Germany in the same year: Statistisches Bundesamt Deutschland, 2013). For further information on this sample see Lüke et al. (2019) and Lüke et al. (2017b).

### Design and Procedure

This longitudinal study incorporates a total of 14 observation sessions over the course of 5 years (1;0, 1;2, 1;4, 1;6, 1;9, 2;0, 2;6, 3;0, 3;6, 4;0, 4;6, 5;0, 5;6, and 6;0; cf. Lüke et al., 2017a, b, 2019). Here, we refer to data from across the full time-period of data collection, specifically to data from 1;0, 3;0, 5;0, and 6;0 years of age.

#### Eliciting and Coding of Pointing Gestures

The gestural behavior of the children at 1;0 was captured in a semi-natural setting within a room equipped with 16 interesting objects and pictures (cf. Liszkowski and Tomasello, 2011). Caregivers were instructed to engage with their children while carrying them for 6 min and to look at various items without touching them. Caregivers were not aware of the aim of the study and the fact that gestures were being analyzed. Four cameras recorded the scene from the four corners of the room. Two research assistants coded the videos for the occurrence of pointing using the annotation tool ELAN (EUDICO Linguistic Annotator; Lausberg and Sloetjes, 2009). Pointing was defined as an extension of the hand and the arm at least more than halfway toward an object or picture without grabbing or touching it. Pointing gestures were coded either as index-finger points—when the index finger was clearly extended relative to all other fingers—or as hand points—when the index finger was not clearly extended relative to the other fingers. Based on this procedure, children were classified as index-finger pointers if they pointed at least once with the index finger or as hand pointers if they did not point with the index finger (cf. Liszkowski and Tomasello, 2011).

To assess interrater reliability, a random 10% of the collected data was coded twice and independently by the two coders. With Krippendorff′s α = 0.968, the interrater reliability for infants’ pointing was very good (cf. Krippendorff, 2013).

#### Measuring Comprehension of Iconic Gestures

The comprehension of iconic gestures was measured when the children were 3;0, 4;0 and 5;0, using an *iconic gesture test* that was developed for this study. The iconic gesture test consisted of 16 iconic signs from German Sign Language (Deutsche Gebärdensprache, DGS; Maisch and Wisch, 1994-2001) as test items and one further item for explaining and practicing the procedure (Table 1). The children were required to match each iconic sign to one of four colored drawings. Comparable to Tolar et al. (2008) the iconic signs were selected based on their iconicity and sign type. Initially, 20 signs that were perceived to be iconic, half categorized as perceptual and half as pantomime (cf. Tolar et al., 2008) were selected. Iconicity was determined based on ratings of 38 university students without any knowledge of sign language. The students were asked to match one of the four drawings to the presented signs. For 17 items, 100% of the students matched the correct drawing to the sign, so that these 17 items (16 test items and one training item) were chosen for the iconic gesture test (Table 1). The 16 test items were randomly ordered, and the target drawings were equally distributed between the four positions on the pages. The experimenter explained the procedure to the child (original in German, here translated in English): “Look, I show you some movements and you show me which of these pictures match with the movement. For example, show me [GESTURE].” For the training item, the children received feedback: in case of a correct answer for the training item: “Yes, that is correct. This [GESTURE] matches with a hat.” and in case of an incorrect answer: “No. Look, this [GESTURE] matches best with a hat (pointing to the picture of the hat).” For the test items, the experimenter gave no feedback regarding the children’s answers. The testing was videotaped, and the performances of the children were rated as either correct or incorrect based on these videos.

**TABLE 1:** Perceptual and pantomime iconic gestures of the iconic gesture test.

Perceptual iconic gestures	Pantomime iconic gestures
Car	Ball
Banana	Tree
Ice cream	Glasses
Comb	Book
Knife	Elefant
Scissors	Window
Key	Cucumber
Telephone	Rabbit
Bird	House
Toothbrush	Cat

#### Measuring Language Development

The language skills of the children were measured at the ages of 1;0, 2;0, 2;6, 3;0, 4;0, 5;0, and 6;0. To do so, two commonly used parent questionnaires (German versions of the MacArthur–Bates Communicative Development Inventories MCDI; Fenson et al., 2007) and various standardized tests were used. Table 2 gives an overview about the parental reports and tests used and the linguistic components addressed. Children were tested individually in a quiet and low-stimulus room.

**TABLE 2:** Parental reports and standardized tests used to measure language skills.

Subtest/inguistic component	Age	1;0	2;0	2;6	3;0	4;0	5;0	6;0
Productive vocabulary size		ELFRA 1	FRAKIS	FRAKIS				
Word comprehension			SETK-2	SETK-2				
Sentence comprehension			SETK-2	SETK-2	SETK 3-5		TROG-D	TROG-D
Word production			SETK-2	SETK-2	PDSS	P-ITPA	P-ITPA	P-ITPA
Sentence production			SETK-2	SETK-2	SETK 3-5			
Grammar production						P-ITPA	P-ITPA	P-ITPA
Sentence repetition						P-ITPA	P-ITPA	P-ITPA

*ELFRA 1: Elternfragbogen für die Früherkennung von Risikokindern (Parent Questionnaire for Early Detection of Children at Risk; German MCDI-version for children at 12 months), Grimm and Doil, 2006; FRAKIS: Fragebogen zur frühkindlichen Sprachentwicklung (Questionnaire for Early Language Acquisition; German MCDI-version for children between 18 and 30 months), Szagun et al., 2009; SETK-2: Sprachentwicklungstest für zweijährige Kinder (Language Acquisition Test for 2-year-old Children), Grimm, 2000; SETK 3-5: Sprachentwicklungstest für drei- bis fünfjährige Kinder (Language Acquisition Test for 3- to 5-year-old Children), Grimm, 2001; PDSS: Patholinguistische Diagnostik bei Sprachentwicklungsstörungen (Patholinguistic Diagnostics for Language Impairments), Kauschke and Siegmüller, 2010; P-ITPA: Potsdam-Illinois Test für Psycholinguistische Fähigkeiten (Potsdam-Illinois Test of Psycholinguistic Abilities), Esser and Wyschkon, 2010; TROG-D: Test zur Überprüfung des Grammatikverständnisses (German version of the Test for Reception of Grammar), Fox, 2013.*

#### Criteria for Language Delay and Developmental Language Disorder

Significant language difficulties were defined as a result of the children’s scores in the standardized language tests. For this reason, the results in at least one language subtest had to be 11/2 SD below the mean (i.e., T score of ≤35) and 1 SD below the mean in at least one additional language subtest presented in Table 2 (i.e., T score of <40). At the ages of 2;0 and 2;6, children who fulfilled these criteria received the diagnosis *language delay* (LD) and at the ages of 3;0, 4;0, 5;0, and 6;0 they received the diagnosis *developmental language disorder* (DLD).

### Data Analysis Plan

Analyses focus on the prediction of language skills at 5;0 and 6;0. Therefore, eight multiple stepwise regression analyses were run with the independent variables: index-finger pointing at 1;0 (dichotomous), non-verbal IQ measured at 3;6 and the SES of the family; and the dependent variables: sentence comprehension, sentence repetition, word production, and grammar production at 5;0 and 6;0. Predictor variables were included if they significantly improved the ability of the models to predict the outcome variables.

Further to this, mediation analyses were performed using the PROCESS macro by Hayes (2018) to analyze whether the predictive value of index-finger pointing at 1;0 for language skills at 5;0 and 6;0 would be mediated by the language skills at 3;0. For the mediation analyses, the results of the three language tests (sentence comprehension, word production, and grammar production) at 3;0, 5;0, and 6;0 were summarized via principal component analyses for each age separately. The Kaiser-Meyer-Olkin measure verified the sampling adequacy for the analyses, KMO_3;0_ = 0.724, KMO_5;0_ = 0.726, KMO_6;0_ = 0.717. At all three ages one factor was extracted, because of their eigenvalues of 2.29 at 3;0, 2.48 at 5;0, and 2.19 at 6;0 being over Kaiser’s criterion of 1. This one factor explained 76.2% of the variance at 3;0, 82.7% at 5;0, and 73.1% at 6;0. For these principal component analyses, missing data were substituted with the mean value. Pairwise deletion was used in all other analyses.

Regarding the relation of early index-finger pointing, iconic gesture comprehension, and language skills Pearson-correlations were calculated. Based on these results, mediation analyses were performed using the PROCESS macro by Hayes (2018) to analyze whether the predictive value of index-finger pointing at 1;0 for language skills at 5;0 and 6;0 would be mediated by the iconic gesture comprehension at 3;0.

For comparing the gesture and language skills of the two groups of children (TD vs. LD) non-parametric tests (Mann-Whitney *U*) were used due to unequal sample sizes. As measure of effect size Pearson’s *r* is reported.

## Results

### The Predictive Value of Index-Finger Points for Language Skills at 5;0 and 6;0

**FIGURE 1:**
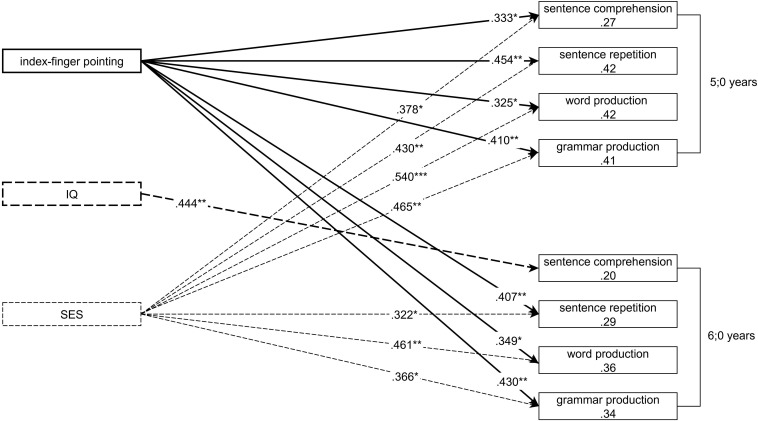
Final models of eight stepwise regression analyses with the independent variables: index-finger pointing at 1;0, non-verbal IQ measured at 3;6 and the SES of the family; and the dependent variables: sentence comprehension, sentence repetition, word production and grammar production at 5;0 and 6;0. Predictor variables were included if they could significantly improve the ability of the models to predict the outcome variables. Presented are standardized betas of the predictor variables and the R^2^ of the final models, *p < 0.05, **p < 0.01, ***p < 0.001. Detailed statistics are presented in the online Supplementary Materials.

The ability to point communicatively with the extended index finger at the age of 12 months is predictive for almost all of the children’s measured linguistic skills and their working memory at 5;0 and 6;0 (Figure 1).

Together with the SES of the family, the production of index-finger points accounts, for example, for 42% of the variance in the productive lexical skills of the children at 5;0 and 36% of the variance in the same competence at 6;0.

**FIGURE 2:**
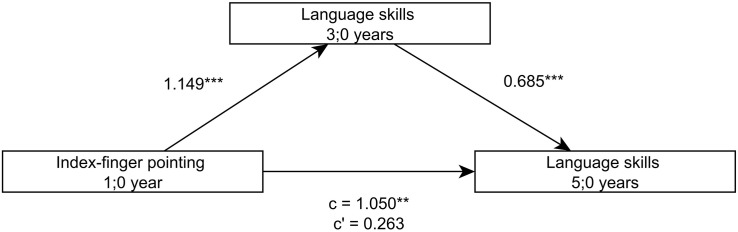
Model of index-finger pointing at 1;0 as a predictor of language skills at 5;0, mediated by language skills at 3;0.

**FIGURE 3:**
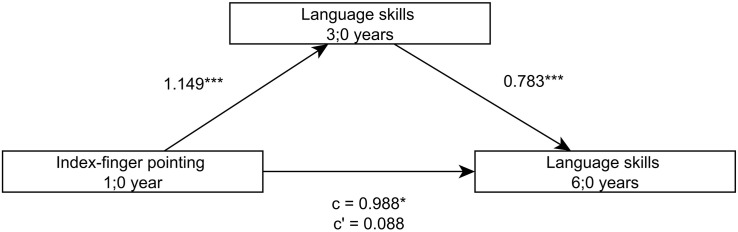
Model of index-finger pointing at 1;0 as a predictor of language skills at 6;0, mediated by language skills at 3;0.

Looking at the language development of the children more closely, further analyses demonstrate that the language skills at 3;0 (sentence comprehension, word production and grammar production), both separately as well as combined via a factor analysis, predict later language skills at 5;0 and 6;0 and that the relation between the index-finger pointing at 1;0 and the later language skills is completely mediated by the language skills at 3;0 (Figures 2, 3).

### Relation Between Comprehension of Iconic Gestures, the Production of Index-Finger Points and Linguistic Skills

The ability to understand the iconicity in gestures was measured when the children were 3;0, 4;0 and 5;0. This ability correlates with earlier and later language skills (Table 3); the comprehension of iconic gestures at 3;0 in particular is strongly related to language abilities as well as to index-finger pointing at 1;0.

**TABLE 3:** Relation between comprehension of iconic gestures, index-finger pointing at 1;0 and language abilities (Pearson-correlation).

		Comprehension of iconic gestures		
	*n*	3;0	4;0	5;0
Comprehension of iconic gestures at 3;0	39	–	0.396*	0.332*
Comprehension of iconic gestures at 4;0	40	0.396*	–	0.501**
Comprehension of iconic gestures at 5;0	41	0.332*	0.501**	–
Index-finger pointing at 1;0	42	0.574**	0.368*	0.396*
Non-verbal IQ at 3;6	39	0.333*	0.458**	0.475**
Word comprehension at 2;0	40	0.499**	0.247	0.353*
Sentence comprehension at 2;0	39	0.478**	0.358*	0.495**
Word production at 2;0	42	0.620**	0.302	0.556**
Sentence production at 2;0	36	0.641**	0.433*	0.521**
Sentence comprehension at 3;0	37	0.542**	0.329	0.332*
Word production at 3;0	39	0.455*	0.307	0.357*
Sentence production at 3;0	38	0.482*	0.332*	0.282
Word production at 4;0	41	0.469**	0.381*	0.282
Grammar production at 4;0	39	0.726**	0.331*	0.328*
Sentence repetition at 4;0	37	0.480*	0.197	0.194
Sentence comprehension at 5;0	41	0.460**	0.282	0.456**
Word production at 5;0	41	0.437**	0.255	0.286
Grammar production at 5;0	41	0.653**	0.317*	0.265
Sentence repetition at 5;0	40	0.455**	0.301	0.209
Sentence comprehension at 6;0	41	0.418**	0.275	0.255
Word production at 6;0	41	0.319	0.310	0.118
Grammar production at 6;0	41	0.643**	0.414**	0.275
Sentence repetition at 6;0	40	0.428**	0.347*	0.208

*n*: Due to the fact that not every child participated in every test, the exact number of participating children is listed; * *p* < 0.05, ** *p* < 0.01, *** *p* < 0.001.

**FIGURE 4:**
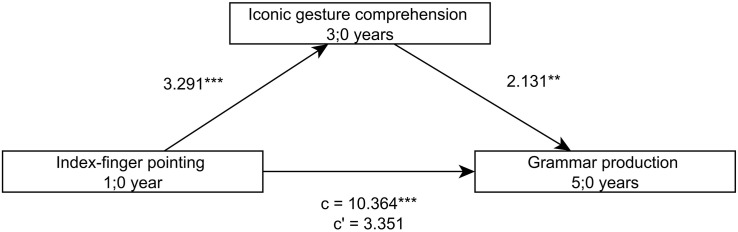
Model of index-finger pointing at 1;0 as a predictor of productive grammar skills at 5;0, mediated by iconic gesture comprehension at 3;0.

**FIGURE 5:**
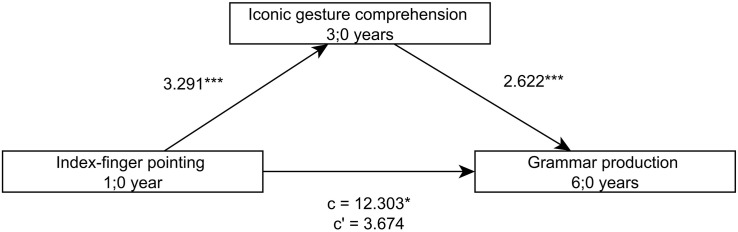
Model of index-finger pointing at 1;0 as a predictor of productive grammar skills at 6;0, mediated by iconic gesture comprehension at 3;0.

Comparable to the language skills of the children at 3;0, the comprehension of iconic gestures at 3;0 mediates the relation between the ability to point with the extended index finger at 1;0 and the grammar skills at 5;0 and 6;0 (Figures 4, 5). Yet, such a mediating effect of iconic gesture comprehension is not found for the relation between index-finger pointing at 1;0 and sentence comprehension or lexical skills at 5;0 and 6;0.

### Gesture and Language Development of Children With LD and DLD

At 2;0, ten children from this sample were identified as language-delayed (Lüke et al., 2017b). The parents of these children were given the option to take part in two sessions with a speech-language pathologist, who explained and demonstrated language beneficial behavior and answered questions of the parents that they might have had about language acquisition. Nine of the ten families agreed to this option so that nine mothers and their children with LD received two sessions with the speech-language pathologist. At the age of 2;6 five of the ten children with LD caught up so that they no longer met the criteria for LD. The parents of the other five children were recommended to begin speech and language therapy (SLT) with their child in the clinic for SLT at the TU Dortmund University. At first, four of the five families started SLT, but one family discontinued the therapy after a few units. The two children who either did not receive or only participated for a very short time in SLT had amasses significant language deficits by the end of the study, when the children were 6;0. At least once a year, the parents of those two children were invited to a talk about their children’s development in which the importance of SLT for their children was highlighted. The parents refused to start SLT, in one case for personal reasons, in the other due to the parents’ diverging assessment of their child’s language skills. Table 4 gives an overview of the language development of the ten children who were identified as language-delayed at 2;0.

**TABLE 4:** Development of 10 children with LD at 2;0.

No.	Sex	Familiy history of LD	Index-finger point at 1;0	Vocabulary at 2;0^a^	Comprehension deficit at 2;0^b^	Parental guidance^c^	Status 2;6	SLT	Status 3;0–6;0
1	Female	No	No	16	No	Yes	LD	10 units	TD
2	Male	Yes	No	23	Yes	Yes	LD	20 units	TD
3	Female	Yes	No	69	Yes	No	LD	No	DLD^d^
4	Female	No	No	123	Yes	Yes	LD	Breakup	DLD
5	Male	No	No	210	Yes	Yes	TD	–	TD
6	Male	No	No	95	Yes	Yes	TD	–	TD
7	Female	Yes	Yes	41	No	Yes	TD	–	TD
8	Female	Yes	No	152	Yes	Yes	LD	10 units	TD
9	Male	No	Yes	28	Yes	Yes	TD	–	TD
10	Male	No	No	3	No	Yes	TD	–	TD

*a = Size of productive vocabulary measured with FRAKIS (Table 2); b = In at least one of the two standardized tests of the SETK-2 (Table 2) measuring word and sentence comprehension a score of >1 SD below the mean; c = taking part in two units with a speech and language pathologist who explained and demonstrated a language beneficial behavior; d = did not participate at 5;0; SLT = speech and language therapy; LD = language delay; TD = typical development; DLD = developmental language disorder.*

**FIGURE 6:**
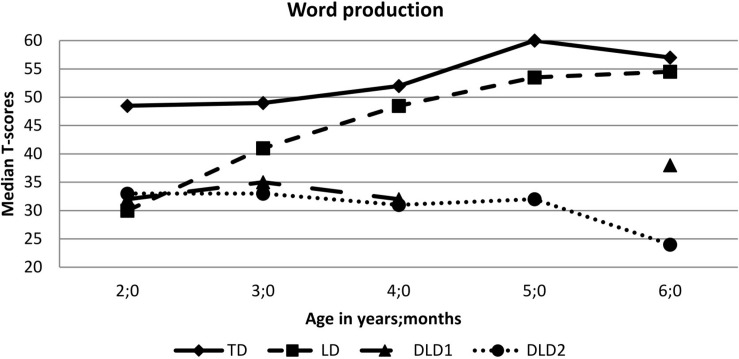
Performances in the word production tasks of children with TD (*n* = 29–32), children with a LD at 2;0 (*n* = 6–8) and two children with a DLD (DLD1 and DLD2) between 2;0 and 6;0.

Comparing the two groups of children, TD children without any language problems at any time and children with LD at 2;0, revealed significantly lower language skills in children with LD at 2;0 all the way through the age of 6;0. These differences between the two groups are highly driven by the results of the two children with the apparent DLD. Excluding the two children with a DLD from the analyses, no differences between TD children and children with a history of LD were found in the word production task (Figure 6) but in the area of sentence comprehension, grammar production, and sentence repetition at 5;0 (Table 5). At the age of 6;0, no more differences in the language skills between the two groups are found when the two children with DLD are excluded from the analyses (Table 5).

**TABLE 5:** Comparison of the language skills of children with and without a history of LD.

		TD (*n* = 29–32)		LD (*n* = 6–10)				
		*Md*	*IQR*	*Md*	*IQR*	*U*	*p*	*r*
5;0	**One child with DLD within the group of children with LD**							
	Sentence comprehension	51.0	9.0	43.0	10.0	70.0	0.019	0.36
	Word production	60.0	11.8	53.0	12.0	81.0	0.047	0.31
	Grammar production	59.0	14.0	53.0	8.0	68.5	0.017	0.37
	Sentence repetition	64.0	12.0	56.0	16.0	63.0	0.013	0.40
	**Children with DLD are excluded from analyses**							
	Sentence comprehension	51.0	9.0	54.5	8.0	67.0	0.038	0.33
	Word production	60.0	11.8	53.5	10.3	81.0	0.111	0.25
	Grammar production	59.0	14.0	53.5	7.8	68.5	0.044	0.32
	Sentence repetition	64.0	12.0	57.0	8.3	63.0	0.033	0.35
6;0	**Both children with DLD within the group of children with LD**							
	Sentence comprehension	55.0	12.0	48.0	12.3	77.0	0.017	0.38
	Word production	57.0	10.0	48.5	19.0	86.0	0.036	0.34
	Grammar production	62.0	16.0	52.5	19.8	80.5	0.023	0.36
	Sentence repetition	65.0	13.0	59.0	21.5	81.5	0.060	0.36
	**Children with DLD are excluded from analyses**							
	Sentence comprehension	55.0	12.0	49.0	10.5	77.0	0.100	0.27
	Word production	57.0	10.0	54.5	15.0	86.0	0.186	0.22
	Grammar production	62.0	16.0	55.0	13.0	80.5	0.130	0.25
	Sentence repetition	65.0	13.0	59.0	25.0	78.5	0.257	0.26

*TD = typical development; LD = language delay; DLD = developmental language disorder.*

Until the age of 5;0 differences in sentence comprehension, grammar production and sentence repetition were found between TD children and children with a history of LD, even when the children with a DLD were excluded from analyses. The same is true for the comprehension of iconic gestures. Children with LD at the age of 2;0 who caught up with or without the help of SLT by the age of 3;0, develop the comprehension of iconic gestures later than children without any history of LD (Table 6).

**TABLE 6:** Comparison of iconic gesture comprehension of children with and without a history of LD.

	TD (*n* = 30–32)		LD (*n* = 7–9)				
	*Md*	*IQR*	*Md*	*IQR*	*U*	*p*	*r*
**Both children with DLD within the group of children with LD**							
3;0	8.0	3.3	5.0	1.5	46.0	0.003	0.52
4;0	12.0	3.0	9.0	1.5	61.5	0.011	0.45
5;0^a^	13.0	2.0	10.0	1.5	36.0	0.001	0.53
**Children with DLD are excluded from analyses**							
3;0	8.0	3.3	5.0	2.0	42.0	0.014	0.46
4;0	12.0	3.0	9.0	1.0	56.5	0.048	0.40
5;0	13.0	2.0	10.0	1.8	31.0	0.001	0.52

*TD = typical development; LD = language delay; DLD = developmental language disorder; a, only one child with a DLD within the group of children with a LD.*

## Discussion

This longitudinal study provided a total of 14 observation sessions, in which the gestural and language abilities of children as well as their communicative behavior within semi-natural interactions with the caregivers were assessed. At the age of 2;0 years, ten of the 42 children were identified as having a LD. Our analysis provides developmental insights into how early performance in gesture production and comprehension is predictive for earlier and later language skills. Below, we will present and discuss our findings according to the goal of this study which was threefold. First, we analyzed the predictive value of early index-finger pointing for language skills beyond the age of 4;0–4;6 (Cartmill et al., 2014; Lüke et al., 2019). The results show that the ability to point with the extended index finger at 12 months is predictive for receptive and productive language skills at 5;0 and 6;0 within the areas of lexicon, morphology, and syntax as well as for the phonological working memory. This is in line with current research (Colonnesi et al., 2010; Murillo and Belinchón, 2012; Beuker et al., 2013; Igualada et al., 2015; Lüke et al., 2017a, 2019), but extend these previous results across preschool childhood. The results of our comprehensive longitudinal study convincingly demonstrate an indirect predictive value of index-finger pointing at 12 months for language skills at 5;0 and 6;0 that is mediated by the language skills of the children at 3;0. Index-finger pointing as one of the first means of intentional communication is related to more advanced verbal communicative skills at 3;0 such as productive lexicon and sentence production and comprehension. At 3;0, these linguistic skills in turn are related to more advanced lexical and syntactic skills 2 and 3 years later.

For the second goal, the relation between early index-finger pointing, iconic gesture comprehension, and later language skills was investigated. We argued that iconic gesture comprehension reflects the children’s ability to select relevant aspects of the referent. We found that children’s understanding of iconic gestures correlated with both, early index-finger pointing and earlier as well as later language skills. Moreover, iconic gesture comprehension was found to be predictive for later grammar skills and mediates the predictive value of index-finger pointing at 1;0 for grammar skills at 5;0 and 6;0.

These first two findings of the strong and long-lasting predictive merit of index-finger pointing at 12 months and also the predictive value of iconic gesture comprehension for later language skills support the view that the communication system is not only a multimodal integrated system (cf. McNeill, 1992) but also develops in an integrated way. Volterra et al., 2017, p. 34) came, based on their research, to the same conclusion and stated that “gesture and speech emerge at about the same time, refer to the same broad set of referents, and serve similar communicative functions.”

The strong relation between gestures and language skills is even more important in children with LD and DLD, who were focused on as the third goal of this study. Children with LD and especially with DLD have limited language competencies, which impact their daily social activities and their educational success (Law et al., 2009; Bishop et al., 2017). They often start to talk later than TD children and have a smaller expressive vocabulary than their unimpaired peers (Desmarais et al., 2008) so they use gestures without speech as a means of communication over a longer period (Lüke et al., 2017b). In accordance with this delay, it is not surprising that in many studies accounting for the gesture use in children with and without language disorders, children with significant language problems produce more gestures than TD children of the same age (Iverson and Braddock, 2011; Mainela-Arnold et al., 2014; Lavelli et al., 2015) but as many as younger, language-matched children (Lavelli et al., 2015). Even in studies in which the rate of gesture productions in children with DLD is not higher than the rate in TD children of the same age, children with DLD use gestures more often in an extending way, i.e., adding new information to the verbal utterance through the gesture (Wray et al., 2017). This indicates that children with DLD rely more on gestures to convey their communicative intentions as well as to understand their communication partners. In the study by Botting et al. (2010), children with DLD performed less well in comparison to their TD peers in a speech–gesture integration task but focused more on the information that was expressed by gesture than by speech. This means that although children with LD or DLD score more poorly in a gesture comprehension task—as it was found by Botting et al. (2010) as well as in our own study—they can benefit from the presentation of iconic gestures during word learning (Ellis Weismer and Hesketh, 1993; Lüke and Ritterfeld, 2014; Vogt and Kauschke, 2017).

The findings of our longitudinal study regarding children with LD and DLD support the current state of research and expand it by showing that children with LD start to produce index-finger points at a later age (Lüke et al., 2017b), use more hand and index-finger points at the end of the second year of life (Lüke et al., 2017b), and develop the ability to understand iconic gestures later than their TD peers. The gestural development of children with LD and DLD does not seem to be entirely different from the developmental pathways in TD children but is time-delayed. This delay in development is supported by studies conducted in comparison to groups of TD children who are of the same age and to language-matched children who are younger; these studies show that the gestural behavior of children with LD or DLD is comparable to the performances of the younger, language-matched children (Lavelli et al., 2015). However, all evidence indicates that speech and gesture form an integrated communication system that functions similarly in children with and without language problems. Facing these similarities, the question appears how to explain the benefits of iconic gestures presentation for word learning in both groups, young TD children, and children with LD or DLD (e.g., McGregor et al., 2009; Lüke and Ritterfeld, 2014; Vogt and Kauschke, 2017; van Berkel-van Hoof et al., 2019). Similar results were found for *language* interventions administered by trained parents or by speech-language pathologists (Law et al., 2009; Roberts and Kaiser, 2011): Children with LD and DLD score lower on language and gesture tasks, but they also benefit from the presentation of *more* and *specific* language and gesture input. Parents seem to be sensitive to this effect because they do not only intuitively adapt their verbal input toward their child with LD, as it has been found in many studies (for a review see Blackwell et al., 2015), but also their gestural input (Grimminger et al., 2010; Lavelli et al., 2015; Wray and Norbury, 2018). This means that children with LD or DLD can benefit from the presentation of iconic gestures during word learning as well as they benefit from SLT, although their gestural and language abilities are lower compared to TD peers. Beneficial effects of gesture and language interventions do not pose a contradiction to the assumption of an integrated communication system.

However, the small number of children with LD and DLD in our sample limits the general statements of our study. As stated above, data from large, population-based studies is needed to examine whether the absence of index-finger pointing at 12 months is a valid indicator of a LD. A further limitation is that with the advancing age of the children, we only investigated their iconic gesture comprehension but have no test on their iconic gesture production—a communicative ability that was considered as relevant for language acquisition in other studies (e.g., Botting et al., 2010; Wray et al., 2017). We decided to capture iconic gestures skills in a way which would be least associated with language skills and followed the procedure of Tolar et al. (2008) instead of other procedures which focus directly on the competence to integrate gesture and speech (Botting et al., 2010; Sekine et al., 2015; Wray et al., 2016). We did so, because Botting et al. (2010) and Wray et al. (2016) already showed that children with DLD perform worse in speech–gesture integration tasks compared to TD children. Still, further information on (iconic) gesture development would have been helpful since some questions remain: Although the comprehension of iconic gestures is related to earlier and later language skills and to early index-finger pointing, why is it only predictive for later grammar production skills? Other aspects of iconic gesture comprehension, as measured with speech–gesture integration tasks (Botting et al., 2010; Sekine et al., 2015) or iconic gesture production tasks, could support our understanding of specific relations between gestural and verbal competencies and should be used in further longitudinal studies.

Despite the limits of our study, the strong relation of gesture and language acquisition might be helpful for early identification of children with high risk for LD. In further research, the possibility of using early gestural competencies as a valid screening instrument for LD should be addressed in a large, representative sample including mono- and bilingual children. The method of capturing the gestural abilities of the children should be analyzed systematically in such a study, since methods vary widely.

For research on multimodal communication, our findings raise the necessity to be aware not only of individual differences but also of a subgroup of children who is likely to be a part of any sample in longitudinal studies, namely children with LD or DLD. In contrast to current practices averaging children’s performance and thus generalizing findings to a typical pattern of behavior in developmental studies, we suggest to pay greater attention to this subgroup or individual children performing low on language. We argue that with this special attention, research can reveal a more comprehensive picture on the developmental paths of language and gesture. Moreover, in research focusing on children with LD and DLD inclusion criteria are crucial for the value of predictive variables: For example, the epidemiologic study by Reilly et al. (2018) shows that classifying children as LD (in their study: late talkers) merely based on their productive vocabulary size is not sufficient to reliably predict their ongoing language development. Based on another finding revealing that poor receptive language skills do strongly predict the subsequent language skills in children with LD (Desmarais et al., 2008), we classified children in our study on the basis of multiple standardized language tests, measuring receptive and productive language skills.

In essence, our study provides important insights into the longitudinal development of the communicative system that integrates gestures and speech. In line with the majority of the published studies in this field, our results support the view that gesture and speech form an integrated communication system from early on and that this system functions comparably in children with and without LD or DLD, but with a time delay.

## Data Availability Statement

The datasets generated for this study are available on request to the corresponding author.

## Ethics Statement

The studies involving human participants were reviewed and approved by Internal Review Board of the University of Münster (2011-517-f-S); Internal Review Board of Bielefeld University (EUB 2015-079). Written informed consent to participate in this study was provided by the participants’ legal guardian/next of kin.

## Author Contributions

CL was responsible for data collection, data analysis, and drafted the first manuscript. All authors designed the study, developed the coding system, interpreted the data and revised the manuscript critically.

## Conflict of Interest

The authors declare that the research was conducted in the absence of any commercial or financial relationships that could be construed as a potential conflict of interest.
