# Supplementary material for: Integrated Communication System: Gesture and Language Acquisition in Typically Developing Children and Children With LD and DLD
Source: Front Psychol. 2020 Feb 4;11:118. doi: 10.3389/fpsyg.2020.00118 (PMC7010863; doi:10.3389/fpsyg.2020.00118)
Supplement: Supplementary file 1 [file Data_Sheet_1.PDF]

Supplementary Material

1 Supplementary Tables

Table 1

Final results of four multiple stepwise regression analyses predicting language skills at 5;0 years

Language skill at 5;0 years		Sentence comprehension	Sentence repetition	Word production	Grammar production
Constant	*B* (*SE*)	44.02*** (3.10)	56.76*** (2.50)	52.65*** (2.01)	51.50*** (2.54)
Index-finger pointing	*B* (*SE*)	8.07* (3.61)	9.91* (2.91)	5.75* (2.35)	9.06*** (2.97)
	β	.333*	.454*	.325*	.410**
IQ	*B* (*SE*)				
	β				
SES	*B (SE)*	4.37* (1.72)	4.48** (1.39)	4.55*** (1.12)	4.89** (1.41)
	β	.378*	.430**	.540***	.465**
R^2		.27	.42	.42	.41
F		6.11**	11.77***	11.97***	11.39***

Note. Final models of four stepwise regressions with the independent variables index-finger pointing at 1;0, non-verbal IQ measured at 3;6 and the SES of the family and the dependent variables sentence comprehension, sentence repetition, word production and grammar production at 5;0 years. Predictor variables were included if they could significantly improve the ability of the models to predict the outcome variables. *$p < .05$, **$p < .01$, ***$p < .001$.

Table 2

Final results of four multiple stepwise regression analyses predicting language skills at 6;0 years

Language skill at 6;0 years		Sentence comprehension	Sentence repetition	Word production	Grammar production
Constant	*B* (*SE*)	21.66*** (10.88)	56.98*** (2.91)	49.71*** (2.50)	51.36*** (3.23)
Index-finger pointing	*B* (*SE*)		9.38** (3.39)	7.26* (2.91)	11.40** (3.76)
	β		.407**	.349*	.430**
IQ	*B* (*SE*)	0.30** (0.10)			
	β	.444**			
SES	*B (SE)*		3.53* (1.62)	4.58** (1.39)	4.62* (1.79)
	β		.322*	.461**	.366*
R^2		.20	.26	.36	.34
F		8.33**	6.62**	9.12**	8.45**

Note. Final models of four stepwise regressions with the independent variables index-finger pointing at 1;0, non-verbal IQ measured at 3;6 and the SES of the family and the dependent variables sentence comprehension, sentence repetition, word production and grammar production at 6;0 years. Predictor variables were included if they could significantly improve the ability of the models to predict the outcome variables. *$p < .05$, **$p < .01$, ***$p < .001$.
